# Education and Decision-Making: An Experimental Study on the Framing Effect in China

**DOI:** 10.3389/fpsyg.2017.00744

**Published:** 2017-05-11

**Authors:** Wen Fan

**Affiliations:** ^1^School of Public Administration, Nanjing University of Finance and EconomicsNanjing, China; ^2^Center for Social Security Study, Wuhan UniversityWuhan, China

**Keywords:** framing effect, decision making, heterogeneity, higher education policy, China

## Abstract

China’s higher education expansion policy has been in effect for almost two decades. Under this policy, a growing number of youths have gained access to higher education, which aims to train students to be more rational. This study examines human rationality at a Chinese college through an experiment based on the risky-choice framing effect. The basic results show no classical framing effect with regard to individual decisions for the entire sample in a benchmark setting. However, when the participants’ roles were manipulated and subsamples were investigated, a significant framing effect was found that appeared to be role-related and that varied by sex. These results help to elucidate evaluations of the effects of China’s higher education policy and may assist in guiding further policy reforms.

## Introduction

People make many decisions and judgments in daily life, the explanations for and predictions of which are often based on the assumption of human rationality. However, as Tversky and Kahneman (1986) stated, “Alternative descriptions of decision problems often lead to different preferences, contrary to the principle of invariance that underlies the rational theory of choice. Violations of this theory are traced to the rules that govern the framing of decisions and to the psychophysical principles of evaluation embodied in prospect theory” (p. S251). This decision bias is well known as a risky-choice framing effect, in which choices involving gains are frequently risk-averse, whereas choices involving losses are frequently evidence of risk-taking. The framing effect is commonly considered to be one of the most severe violations of normative utility axioms and is therefore a strong indicator of irrationality. Thus, individuals’ preferences will reverse or shift (bidirectional or unidirectional framing effects, respectively) when the same problem is framed in different ways. Classic bidirectional framing effects frequently lead to irrational reversals in risk preferences under different framing conditions. However, as Wang (1996b) defined the term, “[the] unidirectional framing effect involves no preference reversal but a shift to a more extreme risk preference… if the predominant preference is unidirectionally risk-averse under both framing conditions, it is even more risk-averse when positively, as opposed to negatively, framed. Similarly, if the predominant preference is unidirectionally risk-seeking under both framing conditions, it is even more so under a negative frame” (p. 5).

This study investigates human rationality through an experiment that examines the risky-choice framing effect. The participants were undergraduate students from two different majors at a Chinese college. Many studies have been conducted with US college students (e.g., Tversky and Kahneman, 1981; Levin et al., 2002). However, recent studies in the Chinese context have been limited by small sample sizes or the lack of a detailed analysis. For example, Zhang and Miao (2008) conducted similar lab experiments, but the authors focused on the experiment *per se*. Zhang et al. (2008) included both military and civilian students, but the authors did not explore heterogeneity effects. Compared to the sample sizes of similar experiments conducted in developed countries, this study’s larger sample size (*N* = 351) yields more power for testing the reliability of the framing effects. In addition, recruiting participants from diverse majors allowed a further exploration of the different individual factors that have been employed as determinants (e.g., self-esteem, numeracy, and biological conditions). Thus, this lab experiment at a Chinese college contributes new evidence to the literature by allowing identification of the perspective- and sex-dependent choice patterns that reveal that human cognitive mechanisms are sensitive to the internal biological status of the information-processing organism.

In addition, as the Chinese Higher Education (HE) expansion policy has been in effect for almost two decades, the public is highly interested in the linkage between this nationwide education policy and the social and economic changes it has brought to Chinese life and society.^1^ There is substantial literature on how education in different disciplines relates to people’s choices (see Chambaere et al., 2013, for end-of-life research; Kidd et al., 2015, for pro-environment behavior analysis; Nafría et al., 2015, for an eParticipatory decision-making study; and Petroman et al., 2015, for food consumption).

Most importantly, there is an increasing body of literature showing that individual rationality or decision-making power appears to be positively related to education level. Anderson et al. (2017) found strong evidence that a husband’s allocation of decision-making authority to his wife varies according to his wife’s age and education. The authors stated that “on average, intra-household accord over which spouse holds decision-making authority is more likely in households where women have higher levels of education” (p.170). From the organizational and financial streams of literature, Mitchell and Lusardi (2015) called for financial education in the workplace, in which they observed that many decisions, such as retirement saving and pension contribution rates, require a certain level of financial literacy. Both Lusardi (2008) and Oehler and Werner (2008) suggested that education could supplement structural pension arrangements such as automatic enrollment. In a recent study that directly investigated how framing effects could be used by HR teams in developing pension structure and communication policies, Maloney and McCarthy (2016) suggested a significant role for personal education and financial literacy in achieving more desirable outcomes and improving the efficacy of such decision-making processes. Arcidiacono (2011, p. 521) claimed that “…it is mainly those who have a lower income or a lower education level who are more likely to fall prey to framing.” In a similar vein, this study examines whether college students’ reasoning increases with a higher level of education, as expected by the Chinese HE expansion policy.

## Materials and Methods

### Participants and Design

**FIGURE 1:**
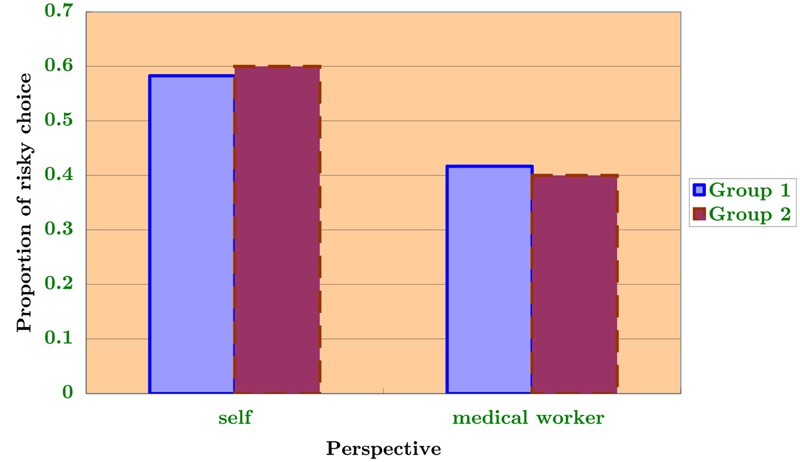
**Counterbalanced orders (positive frame)**.

**FIGURE 2:**
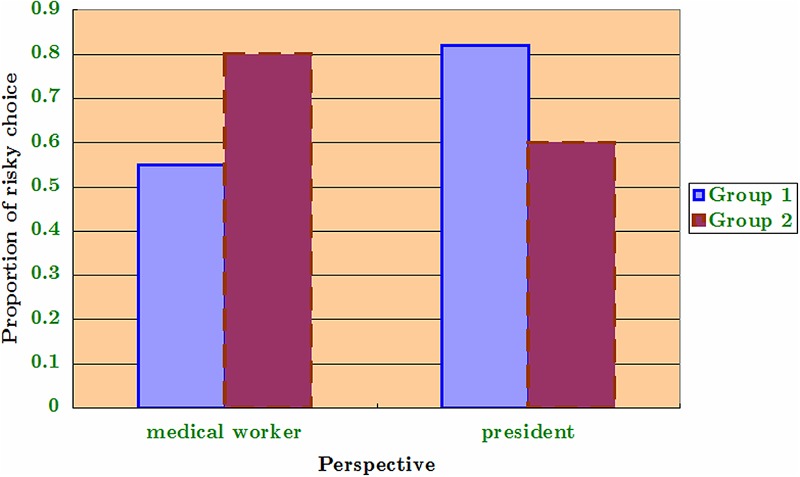
**Counterbalanced orders (negative frame)**.

The participants included 351 students (65% female, female mean age = 21.6, male mean age = 21.8) studying in two majors (mean age = 20 for social science and mean age = 22 for engineering) at a 4-year college in eastern China; the students received class credit in exchange for their participation in the experiment. The responses were anonymous, and the instructions specified that although there was no “correct” answer to the problem, careful thinking would be highly appreciated. The students were not permitted to speak with one another during the experiment. All questions were presented in written form, and the split-ballot questionnaire was administered in two classrooms. The study was approved by the research ethics committee of Nanjing University of Finance and Economics, and conforms to the ethical principles of the Declaration of Helsinki (World Medical Association, 2013). Before administering the experiment, one of the instructors translated the principle of informed consent according to Standard 8.02 of the American Psychological Association’s (APA) new ethics code into Chinese and explained it to all of the participants. The experiment had a two-way mixed design in which an alpha level of 0.05 was used for all *t*-tests. The related measures independent variable was the within-participants manipulated role (general, medical worker, and president). The unrelated measures independent variable was the between-participants decision-making domain (gain and loss). Following Harris (2008), a pilot test was administered on a randomly selected smaller group that allowed the order of roles to be counterbalanced such that their order of presentation varied among the participants. Trial 1 was under the positive frame, in which one group of subjects (Group 1) first received the Q2 scenario (imagining themselves as a medical worker) and a second group (Group 2) first received the Q1 scenario (just acting as themselves). The results are shown in **Figure 1**, where the dependent variable is the proportion of risky choices. It is easy to observe that the role shifting to “acting as a medical worker” largely reduced the probability of risk-seeking for both groups, irrespective of the order of the perspectives that they encountered. Trial 2 was under the negative frame, in which one group of subjects (Group 1) received the Q3 scenario (imagining themselves as the president) first and a second group first (Group 2) received the Q2 scenario (imagining themselves as a medical worker). The results are shown below in **Figure 2**, where the dependent variable remains the same. Comparing the two manipulated roles, the results are similar to those of Trial 1. That is, there is no significant order effect actually incurred.

### Materials and Procedure

The current study slightly modified the Asian Disease Problem developed by Tversky and Kahneman (1981) by manipulating the participants’ roles as “a medical worker” and “the president.” It is worth noting that how the participants would respond to such manipulation was somewhat unpredictable, so some assumptions are provided below:

A1: “A medical worker” would provide a sense of professionalism, sympathy and caring in this life-and-death problem that would make the participants more willing to choose the probability of saving everyone over the definite loss of some lives.^2^
A2: “The president” would deliver a sense of power or a “big figure” feeling that may induce participants to consider the problem in terms of the overall (even political) picture.

Students were assigned to Problems 1 or 2 based on their student numbers or seats. Because different majors were involved in the experiment, the group assignment methods differed according to the class size. In the social science class (the smaller class), students were assigned Problem 1 if their student number ended with an odd digit, whereas Problem 2 was assigned to students whose numbers ended with an even digit. In the engineering class (the larger class), Problem 1 was assigned to students who sat in an odd column on the day of the testing, whereas Problem 2 was assigned to those who sat in the even columns. Both selection methods fit the definition of random assignment because the students received random student numbers upon admission, and they were free to sit wherever they wanted on the day of testing with no prior notification of the experiment.

Q1: Imagine that China is preparing for the outbreak of an unusual disease that is expected to kill 6000 people. Two alternative programs to combat the disease have been proposed. Assume that the exact scientific estimates of the consequences of the programs are as follows:
(Problem 1)
If Program A is adopted, 2000 people will be saved.
If Program B is adopted, there is a 1/3 probability that 6000 people will be saved and a 2/3 probability that no people will be saved.
Which of the two programs would you favor?
The second group was provided the cover story for Problem 1 but with different alternatives:
(Problem 2)
If Program C is adopted, 4000 people will die.
If Program D is adopted, there is a 1/3 probability that nobody will die and a 2/3 probability that 6000 people will die.
Which of the two programs would you favor?

The current study also added two new questions not previously used by Tversky and Kahneman (1981) by asking the students to imagine themselves in different roles for further investigative purposes. The Problems and Programs were the same as *Q1*, so they are not listed again here.

Q2: The problem you are facing is the same as above, but now imagine you are a medical worker. Which of the two programs would you favor?
Q3: The problem you are facing is the same as above, but now imagine you are the president. Which of the two programs would you favor?

## Results

### Main Results

For all three questions shown above, it is clear to see the two problems in each question are effectively identical. The only difference between them is that for Problem 1, the outcomes are framed in positive (described as the number of lives saved) terms and for Problem 2, the outcomes are framed in negative (described as the number of lives lost) terms. In the text and tables below, the questions are labeled ‘*Q1*,’ ‘*Q2*,’ and ‘*Q3.*’ The total number of respondents for each problem is denoted by *N*, and the percentage of participants who chose each option is indicated in brackets.^3^

#### *Q1* Scenario

**Problem 1 [*N* = 191]:**

If Program A is adopted, 2000 people will be saved. **[42%]**
If Program B is adopted, there is a 1/3 probability that 6000 people will be saved and a 2/3 probability that no one will be saved. **[58%]**

No risk aversion emerged in this case. By contrast, the participants were more risk-seeking in both the positive (*t* = -4.42, *p* < 0.05) and negative (*t* = -4.53, *p* < 0.05) framing conditions.

#### *Q2* Scenario

**Problem 1 [*N* = 191]:**

If Program A is adopted, 2000 people will be saved. **[45%]**
If Program B is adopted, there is a 1/3 probability that 6000 people will be saved and a 2/3 probability that no one will be saved. **[55%]**

**Problem 2 [*N* = 160]:**

If Program C is adopted, 4000 people will die. **[28%]**
If Program D is adopted, there is a 1/3 probability that no one will die and a 2/3 probability that 6000 people will die. **[72%]**

Notably, the participants’ choices changed to a certain extent when they imagined themselves in the role of a medical worker. They were neither risk-averse nor risk-seeking in response to Problem 1 (*t* = -1.21, *p* > 0.05); however, they continued to exhibit a strong risk-taking tendency in response to Problem 2 (*t* = -17.07, *p* < 0.05).

#### *Q3* Scenario

**Problem 1 [*N* = 191]:**

If Program A is adopted, 2000 people will be saved. **[58%]**
If Program B is adopted, there is a 1/3 probability that 6000 people will be saved and a 2/3 probability that no one will be saved. **[42%]**

**Problem 2 [*N* = 160]:**

If Program C is adopted, 4000 people will die. **[38%]**
If Program D is adopted, there is a 1/3 probability that no one will die and a 2/3 probability that 6000 people will die. **[62%]**

When the participants imagined themselves in the role of president, the results were similar to those found in previous studies; that is, there was a general preference reversal from a predominantly risk-averse choice under a positive frame (*t* = 11.62, *p* < 0.05) to a tiny risk-seeking preference under a negative frame (*t* = -1.89, *p* > 0.05).

The mean differences in risky choices among the three scenarios using positive and negative frames are reported for the full sample in **Table 1**. The results show that there is no framing effect in *Q1* (*t* = -1.63, *p* > 0.05, *d* = 0.30, 95% confidence interval (CI) = [-1.11, 1.68]) and a strong risk-seeking unidirectional framing effect in *Q2* (*t* = -2.94, *p* < 0.05, *d* = 2.04, 95% CI = [-0.20, 3.77]).

**Table 1:** Mean difference in Q1–Q3 by frame.

*N* = 351	*M* positive	*M* negative	*M* positive–*M* negative (framing effect)	*t*	*p*
Q1	0.585	0.692	-0.107	-1.63	0.147
Q2	0.548	0.721	-0.173	-2.94^∗^	0.022
Q3	0.417	0.615	-0.198	-2.28	0.057

*^∗^p < 0.05.*

Notably, a mild bidirectional framing effect appears in Q3 when the participants imagined themselves to be the president (*t* = -2.28, *p* = 0.06, *d* = 0.94, 95% CI = [-0.57, 2.39]), suggesting that the students’ choices were somewhat irrational in that case because “acting as the president” is similar to a daydream and is rather removed from reality. This result occurred because the consequences of life-or-death decisions made by a medical worker or the president might be thought to be different from those made by the participants themselves in terms of the influence of acting in a certain role. This adaptive information makes the subject’s risk proclivity irrational, and the framing effect is thus more likely to be observed in this context. This result, called the perspective-specific risk preference, is compatible with prior findings involving human reasoning (see Gigerenzer and Hug, 1992).

### Sex Differences

To examine the heterogeneity effects, the sample was divided by sex, and the corresponding results are reported in **Table 2**.^4^ As expected, the framing effects tended to vary by sex and remained role-related. There appears to be a significant sex difference for all three manipulated roles under study. The male students did not show a decision bias for either being themselves or being a medical worker (*t* = -1.24, *p* > 0.05, *d* = 0.36, 95% CI = [-2.31, 1.66]) until their role was manipulated to that of the president, a role that is assumed to be more responsible and influential (*t* = -3.27, *p* < 0.05, *d* = 2.5, 95% CI = [-0.57, 5.41]). Notably, this result shows a considerable bidirectional framing effect of risk-seeking, implying that the male students’ judgments were greatly influenced and that there was an increase in ambiguity concerning the choice problem when they imagined themselves to be president. This result might be explained as it was in Wang’s (1996b) study: “If subjects do not really care whether they choose a sure outcome or a gamble, then a minor variation in wording or phrasing may greatly influence their choices” (p. 11).

**Table 2:** Mean difference in Q1–Q3 by frame and sex.

Male					
***N* = 122**	***M* positive**	***M* negative**	***M* positive–*M* negative (framing effect)**	***t***	***p***

Q1	0.552	0.671	-0.119	-1.24	0.303
Q2	0.674	0.728	-0.054	-1.24	0.303
Q3	0.4	0.663	-0.263	-3.27^∗^	0.047

**Female**					

***N* = 229**	***M* positive**	***M* negative**	***M* positive–*M* negative (framing effect)**	***t***	***p***

Q1	0.599	0.817	-0.219	-12.78^∗^	0.001
Q2	0.492	0.677	-0.185	-12.61^∗^	0.001
Q3	0.425	0.324	0.101	1.19	0.321

*^∗^p < 0.05.*

By contrast, female students showed a significant framing effect when both being themselves and a medical worker (*t* = -12.78, *p* < 0.05, *d* = 5.66, 95% CI = [0.35, 11.15]; *t* = -12.61, *p* < 0.05, *d* = 6.1, 95% CI = [0.45, 11.99]). In particular, decisions were highly biased by the bidirectional framing effects of risk-seeking when they imagined themselves in the role of a medical worker, rather than as the president (*t* = 1.19, *p* > 0.05, *d* = 0.14, 95% CI = [-1.84, 2.09]). This result implies that there is a sex difference in the sensitivity to adaptive information embodied in the decision problem. Men tend to be more influenced by a word that reflects certain masculine traits, such as aggressiveness (as is the case when they are acting in the role of president), whereas women pay more attention to words that represent sympathy and caring (as is the case when they are acting in the role of a medical worker).

## Discussion

The discussion is twofold. First, the main results are interpreted. Second, the heterogeneity effects are examined.

As discussed above, no framing effect was found in *Q1*, but a strong unidirectional framing effect was found in *Q2*. How should one interpret this distinction? There are at least two reasons why no framing effect was found in *Q1*. First, Chinese students generally have strong computing skills that are developed in the primary educational system^5^; the respondents in this study were particularly well trained in college economics and finance courses. These features make their risk preferences somewhat unambiguous such that they are more immune to the framing manipulations, which is consistent with the previous literature—Peters and Levin (2008, p, 441) found that “individuals lower in numeracy were influenced more than those higher in numeracy by the frame in the risky-choice scenarios.” Furthermore, the present study can explore the changes being affected in the current Chinese higher education system by comparing this result to that of a similar experiment conducted with Chinese military students who had received less mathematical training during college. This group can serve as a control group in this situation. In that work, Zhang et al. (2008) found that the military participants were influenced by both positive and negative framing. It should be noted that there might be some symmetrical differences between participants with different majors: recent research has reported a relatively low return for engineering higher education in China (Fan and Zhang, 2015). All of these issues call for further study that links the education policy to individual cognition and decision-making through this channel.

Second, the framing effects may be attenuated or offset because of the diverse composition of the entire sample. In other words, human risk preferences and choice strategies are sensitive to a decision-maker’s biological conditions (such as age and sex) as well as to the adaptive values inherent in the choice options (Wang, 1996a). However, a strong unidirectional framing effect was found in *Q2* (when participants imagined themselves as medical workers) because a decision-maker may become more risk and variance seeking to maximize the probability of achieving a goal when the mean expected value is below her minimum requirement (Wang, 1996b). In this life-and-death problem, the minimum requirement tends to be higher than the mean expected value of the choice option—the probability of saving everyone is preferred over the definite loss of some lives.

With regard to sex differences, the present research supports a number of existing studies. Hasseldine and Hite (2003) found no evidence of a main effect for framing objectively equivalent information; however, a significant frame through a gender interaction effect was documented. They reported that “females were more compliant in response to a positively framed persuasive communication than they were to the negatively framed message, even though the information in each message was objectively equivalent” (pp. 528–529). Saad and Gill (2014) showed that women have greater sensitivity to negatively framed information than men do. This study supports the latter finding by showing that more than 80% of the female students selected the risky choice in the negative frame in Q1. Additionally, for female participants only, a conversion from unidirectional framing effects to bidirectional framing effects associated with the perspective shift from oneself to a medical worker was observed. This finding is consistent with Huang and Wang (2010), who argued that the prior design that focused on overall sex differences might be misleading because sex differences in framing effects might vary across different domains. Overall, the present study echoes and emphasizes the relationship between decision-making and gender, which is an interesting topic that is growing in popularity and receiving increased attention in various fields (e.g., Mendelberg and Karpowitz, 2016; Alwang et al., 2017).

## Conclusion

Conventional empirical evidence seems to lead to the conclusion that there are no substantial differences in the rationality of performance when considering variables such as gender, age, education or social status, which is partly due to a lack of standardization and regularity in the methodologies used (e.g., Gigerenzer and Murrey, 1987; Hertwig and Ortmann, 2001, 2005). This experimental study examined the framing effect of Chinese college students with the aim of evaluating the effectiveness of HE reform from a different perspective. The results show that the participants’ preferences were basically unbiased and at least partially driven by the high numeracy skills the participants had accumulated through higher education, which demonstrates the weakness of classical framing effects. Thus, this study offers new evidence on the relationship between education and behavior in China. Furthermore, this study sheds light on how such an analysis might help to elucidate evaluations of the effects of China’s HE policy, under which more young people have access to higher education and receive better training in thinking and reasoning skills. China’s educational system has clearly been expanding on a sustainable developmental path.

When the student responses were sensitively manipulated, however, dividing the sample by sex revealed a tendency for them to be influenced by framing effects. A significant gender difference emerged in this context, which calls for more research to deeply determine whether the framing effects are susceptible to individual differences and sheds light on improving teaching output. More consideration should therefore be given to teaching styles and material as well as students’ aptitudes in light of the sex differences revealed in sensitivity to the adaptive information embodied in the decision problem. Notably, this finding coincides with the doctrine of the Chinese philosopher and educator Confucius, one of whose well-known dictums is “teach students in accordance with their aptitude.” This study may assist in efficiently guiding further policy reforms in this direction. In addition, the findings also imply that the subject’s perspective is an important factor in the decision-making process.

Finally, one potential issue is that the participants may have only provided their estimation of how “certain figures” would respond to each scenario. The participants may lack adequate knowledge of these manipulated roles, which might undermine the results. In this regard, more preliminary work could be conducted in an attempt to improve the research design in the future.

## Author Contributions

WF designed and carried out experiments, as well as analyzed the results and wrote the manuscript.

## Conflict of Interest Statement

The author declares that the research was conducted in the absence of any commercial or financial relationships that could be construed as a potential conflict of interest.

## Footnotes

1 Fan et al. (2015) provided supportive evidence regarding the effect of an HE expansion policy that works through a spillover channel on individual income.

2 People might be concerned about how certain figures such as nurses are trained in China, and in turn whether the level of training would affect the results. In China, after finishing compulsory schooling (graduating from middle school), some students, often those who failed to gain acceptance to a higher-level school, may choose a school of nursing and subsequently take a nursing job. Most such students would never receive higher education. Unlike many developed countries (e.g., the US), nursing education in China is still under-developed in terms of structure, curriculum and faculty training, and the challenges are enormous for Chinese nursing education to meet international standards (Xu et al., 2000). Some recent studies have indicated that although the scale of nursing education at three levels to enter nursing (baccalaureate degree, advanced diploma and secondary diploma) has expanded rapidly in recent years, the current nursing students are still lacking in terms of their education and training levels (You et al., 2010).

3 Notably, the different assignments resulted in relatively large differences between the sample sizes of the two groups (191 students for Problem 1 vs. 160 students for Problem 2).

4 It would also be interesting to divide the sample by major. However, the sample size difference (304 vs. 45) is so extreme that there can be little confidence in the results. Increasing the sample size of social science majors in a future experiment would allow for further examination of this issue.

5 Shanghai has topped two consecutive rounds of the Programme for International Student Assessment (PISA) tests in reading, mathematics and science. The Organization for Economic Cooperation and Development (OECD) administers these tests to assess how well 15-year-olds have acquired the knowledge and skills needed to fully participate in knowledge-driven societies (OECD, 2009, 2012). In addition, a direct measure of numeracy has been designed and will be used in the next experiments.
